# Effect of l-phenylalanine on PAL activity and production of naphthoquinone pigments in suspension cultures of *Arnebia euchroma* (Royle) Johnst

**DOI:** 10.1007/s11627-012-9443-2

**Published:** 2012-06-21

**Authors:** Katarzyna Sykłowska-Baranek, Agnieszka Pietrosiuk, Marcin R. Naliwajski, Anna Kawiak, Małgorzata Jeziorek, Sylwia Wyderska, Ewa Łojkowska, Ioanna Chinou

**Affiliations:** 1 Department of Biology and Pharmaceutical Botany, Faculty of Pharmacy, Medical University of Warsaw, ul. Banacha 1, 02-097 Warsaw, Poland; 2 Department of Plant Physiology and Biochemistry, University of Łódź, ul. Banacha 12/16, 90-237 Łódź, Poland; 3 Department of Biotechnology, Intercollegiate Faculty of Biotechnology, University of Gdańsk–Medical University of Gdańsk, ul. Kładki 24, 80-822 Gdańsk, Poland; 4 Laboratory of Human Physiology, Faculty of Health Sciences with Subfaculty of Nursing, Medical University of Gdańsk, ul. Tuwima 15, 80-210 Gdańsk, Poland; 5 Department of Pharmacognosy, School of Pharmacy, University of Athens, University Campus of Zografou, 157 71 Zografou Athens, Greece; 6 Division Faculty of Biology and Environmental Sciences, Cardinal Stefan Wyszyński University in Warsaw, Dewajtis 5, 01-815 Warsaw, Poland

**Keywords:** *Arnebia euchroma*, l-phenylalanine, PAL activity, Shikonin derivatives, Cytotoxic activity, Secondary metabolites

## Abstract

The effects of l-phenylalanine (PHE) on cell growth and production of shikonin and its derivatives, acetylshikonin (ACS) and isobutyrylshikonin (IBS), in suspension cultures of *Arnebia euchroma* were examined. Supplementing media using PHE have been successfully utilized to enhance shikonin production in cell cultures of other species of Boraginaceae. l-Phenylalanine, the key compound in the phenylpropanoid pathway, is converted by phenylalanine ammonia lyase (PAL) to *trans*-cinnamic acid, which is the precursor of p-hydroxybenzoic acid (PHB). Coupling of PHB and geranyl pyrophosphate (derived from mevalonate pathway) by *p*-hydroxybenzoate-*m*-geranyltransferase leads later to biosynthesis of shikonins. The addition of 0.01 or 0.1 mM PHE to the culture medium stimulated cell proliferation, where the highest observed increase in fresh cell biomass (measured as a ratio of final weight to initial weight) was 12-fold, in contrast to an eightfold increase in control cultures. Whereas, growth media supplemented with 1 mM PHE markedly reduced the rate of cell growth (to only twofold). Precursor feeding had detrimental effects on both ACS and IBS production in all PHE-supplemented media. The highest total content (intracellular + extracellular) of the investigated red pigments (9.5 mg per flask) was detected in the control culture without PHE. ACS was the major component of the naphthoquinone fraction determined in cells and post-culture media. Shikonin itself was found only in the post-culture media from cultures supplemented with 0.01 or 0.1 mM PHE. Increases in PAL activity corresponded well with the accumulation of investigated naphthoquinones in control culture. However, peak PAL activity did not directly correlate with maximum production of shikonin derivatives. Cytotoxicity of extracts, prepared from the cells cultivated in the presence of PHE or in control cultures, was tested on three cancer cell lines: HL-60, HeLa, and MCF-7. The extracts prepared from the untreated control cultures proved to be the most potent against the examined cancer cell lines. The mean inhibitory concentration values were 0.3, 13, and 8 μg ml^−1^ for the HL-60, HeLa, and MCF-7 cells, respectively.

## Introduction

Shikonins and related naphthoquinone compounds are natural lipophilic red pigments occurring in the many plant species belonging to the Boraginaceae family. These compounds are potent pharmaceutical substances that have been shown to elicit significant biological activity including wound healing, antimicrobial, anti-inflammatory, antioxidant, anticancer, and antithrombotic effects (Papageorgiou *et al*. 1999, 2006). They also act as inhibitors of tumor-induced angiogenesis (Hisa *et al*. 1998; Pietrosiuk *et al*. 2004a), and immunomodulatory effects of shikonin derivatives have been also reported (Pietrosiuk *et al*. 2004b). For example, acetylshikonin administered at a 40-μg daily dose stimulated graft-versus-host reaction, but inhibited it at a 200-μg dose. Isobutyrylshikonin at 40-μg doses significantly increased humoral response in the BALB/c mice strain (Pietrosiuk *et al*. 2004b). Shikonin derivatives have been also found to play a role in protecting the immune organs from damage and reversing or enhancing immune responses. In addition, shikonin derivatives improve the quality of life of tumor-bearing mice. Thus, shikonin has potential to be used as an anticancer drug (Su *et al*. 2011).

Shikonin was the first secondary metabolite produced on commercial scale via plant tissue culture (Papageorgiou *et al*. 1999) and is valued at US$4,000 kg^−1^ (Manjkhola *et al*. 2005). Various preparations that contain alkannin/shikonin and derivatives, isolated from the traditional medicinal herbs of *Arnebia euchroma* and *Lithospermum erythrorhizon* of the Boraginaceae family, are still used today for medicinal purposes in China, Japan, and Korea. Moreover, these compounds are also used in Europe, North America, and Japan for cosmetics and dyestuffs.

*A. euchroma* (Royle) Johnst. (Boraginaceae) is a perennial plant of the alpine region distributed in the Pamirs, Tien Shan mountains, and the Himalaya and western Tibet in an altitudinal range of 3,700–4,200 m above sea level (Manjkhola *et al*. 2005). The roots of *A. euchroma* are rich in naphthoquinone compounds, such as alkannin, shikonin, and their derivatives. In comparison to *L. erythrorhizon*, *A. euchroma* is much richer in red pigments and is regarded as an even better source of shikonin-related compounds. The average total content of naphthoquinone pigments in the roots of *L. erythrorhizon*, calculated as alkannin, was 0.47 % (Tang and Eisenbach 1992), while the pigment content in dry mass of *A. euchroma* varied from 0.43 to 2.47 % (Zakhlenjuk and Kunakh 1998).

Roots of *A. euchroma* continue to be harvested from natural sources, contributing to a significant decline in natural plant populations and effectively endangering the species. Biotechnology as well as plant cell culture technology, on the other hand, can be an abundant, novel source of plant-derived compounds (Schürch *et al*. 2008), thereby facilitating conservation and sustainable use of the species. *A. euchroma* tissue culture was pioneered in Russia (Davydenkov *et al*. 1991), and two protocols for *in vitro* organogenesis and embryogenesis have been developed since that provide efficient regeneration systems, necessary for both propagation and preservation of the species (Jiang *et al*. 2005; Manjkhola *et al*. 2005). *Arnebia* sp. *in vitro* culture research has been widely dedicated to improving shikonin production by application of various strategies, among them changing medium composition, addition of different elicitors, and various *in situ* extraction methods (Fu and Lu 1999; Ge *et al*. 2006; Chaudhury and Pal 2010; Kumar *et al*. 2011; Malik *et al*. 2011; Shekhawat and Shekhawat 2011).

**Figure 1.**
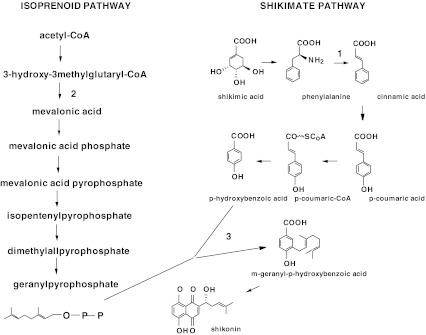
Shikonin biosynthesis pathway adapted from Gaisser and Heide (1996). *1* l-Phenylalanine ammonia lyase (PAL), *2* HMG-CoA reductase, *3* PHB geranyltransferase.

Shikonin and its derivatives are biosynthetically derived from two precursors: 4-hydroxybenzoate formed from l-phenylalanine (PHE) and geranyldiphosphate originating from mevalonic acid (Fig. 1). It has been demonstrated that l-phenylalanine administered to cell cultures of *L. erythrorhizon* was rapidly incorporated into deoxyshikonin and then into fatty acid esters of shikonin. Further investigations revealed that shikonin derivatives are biosynthesized from deoxyshikonin by hydroxylation and esterification at the C-1 position of its side chain (Okamoto *et al*. 1995). Different concentrations of PHE in the medium stimulated the production of shikonin and its derivatives in *Lithospermum* callus cultures (Mizukami *et al*. 1977). Moreover, the inhibition of phenylalanine ammonia lyase (PAL, EC 4.3.1.5) completely suppressed the formation of acetylshikonin (ACS) and shikonin in suspension cultures of *L. erythrorhizon* (Gaisser and Heide 1996).

In the present study, we are the first to report the effects of various l-phenylalanine supplementations on cell growth and the production of shikonin derivatives, and its relationship to PAL activity, in a time-dependent manner. Also, the cytotoxic effects of the obtained *A. euchroma* extracts on three cancer cell lines were examined.

## Materials and Methods

### Cell suspension culture.

Cell suspension cultures of *A. euchroma* (Royle) Johnst. were established from callus tissue cordially donated by Dr. Urmantseva from the All-Russian Institute of Medicinal Plants, Moscow, Russia. Callus was initiated from dormant bud meristems of plants collected in Tajikistan (the western Pamirs) in 1987 (Pietrosiuk *et al*. 1999). The callus cultures were maintained in darkness and subcultured every 4 wk on a solid murashige and skoog for arnebia (MSA) medium (Davydenkov *et al*. 1991). The composition of the MSA medium was (in milligrams per liter): KNO_3_, 3,800; MgSO_4_·7H_2_O, 370; CaCl_2_·6H_2_O, 440; KH_2_PO_4_, 170; FeSO_4_·7H_2_O, 27.8; Na_2_EDTA·2H_2_O, 27.8; MnSO_4_·4H_2_O, 37.3; ZnSO_4_·7H_2_O, 22.3; Na_2_MoO_4_·2H_2_O, 8.6; CoCl_2_·6H_2_O, 0.2; H_3_BO_3_, 0.025; KI, 6.2; CuSO_4_·5H_2_O, 0.83; thiamine, 0.3; pyridoxine, 0.4; and nicotinic acid, 0.5. The MSA medium was supplemented with 1 mg l^−1^ kinetin and 0.3 mg l^−1^ indole-3-acetic acid prior to autoclaving at 121°C for 20 min. The cell suspension cultures were housed in 250-ml modified Erlenmeyer flasks containing 50 ml of MSA liquid medium. The modified flasks had two concavities on the opposite sides of the vessel which promoted aeration and reduced cell aggregation. The cell suspension cultures were kept at 25°C in the dark on an INFORS AG TR 250 shaker (Bottmingen, Switzerland) at 105 rpm. Every 4 wk, 1.5 ± 0.05 g fresh weight (FW) of cell aggregates was subcultured into fresh liquid MSA medium.

### Determination of growth and l-phenylalanine treatment.

The time course of cell suspension growth and the effect of PHE on biomass increase and production of shikonin derivatives were examined. Cells weighing 1.5 ± 0.06 g FW were placed into 50 ml MSA liquid medium either supplemented with 1.0, 0.1, or 0.01 mM of PHE, or without PHE (control). PHE was added to the media prior to autoclaving. Starting from day 4 up to day 35 of culture, samples from two flasks for each culture treatment were harvested every 3 or 4 d. The biomass increase, measured both as fresh and dry weight, was recorded. Cells and media from collected samples were separated using a Büchner funnel. Then, cells were gently pressed on filter paper to remove excess medium and weighed. Samples were lyophilized and their dry weight was recorded. The post-culture media were filtered and the pH of the media (pH-METR 5170, Elwro, Wrocław, Poland; Meratronik V 628, Warsaw, Poland), their conductivity (Elmetron, Zabrze, Poland), and sucrose levels (refractometer PZO RP 10, Warsaw, Poland) were determined.

The experiments were conducted three times and two flasks were sampled at each time point. All the data shown are mean ± SD from six cultivation vessels. The results were analyzed using STATISTICA PL 8 (StatSoft, Krakow, Poland).

### Chemical analysis.

Shikonin derivatives were extracted from both the cells and culture media. For obtaining dye fractions, a powdered sample of lyophilized cells was sonicated with n-hexane. The extraction was done for 15 min at 40°C until the red coloration had faced. The media samples were similarly extracted with n-hexane. The extracts were evaporated from the extract solution under reduced pressure. Dry residue was dissolved in methanol and analyzed in a DIONEX HPLC system (Sunnyvale, CA), equipped with an automated sample injector (ASI-100), and UVD 340S detector using the following conditions: gradient elution—acetonitrile (40–0 ml) + 0.04 M orthophosphoric acid (60–100 ml); flow rate, 1.5 ml min^−1^; column, EC 250/4.6 Nucleosil 120–127 mm C18 (Macherey-Nagel, Düren, Germany), and monitoring eluent at 215, 278, 514, and 320 nm. Shikonin (Wako, Tokyo, Japan) and its two derivatives ACS and isobutyrylshikonin (IBS), isolated previously from natural roots of *Lithospermum canescens* (Pietrosiuk and Wiedenfeld 2005), were used as standards and analyzed under the same conditions. Peaks were assigned by spiking the samples with the standards and comparison of the retention times and UV spectra.

### PAL activity assays.

PAL activity was measured according to Zucker (1965), with some modifications. The cells were homogenized in an ice-cold mortar with 5 ml 0.05 M Tris–HCl buffer pH 8.0 containing 0.8 mM β-mercaptoethanol and 1 % *w/v* polyvinylpolypyrrolidone. The homogenate was centrifuged (18,000×*g* for 15 min at 4°C) and the supernatant was used to measure PAL activity. The enzyme reaction mixture consisted of 1 ml 0.05 M Tris–HCl buffer pH 8.0, 0.1 ml enzyme extract, 0.5 ml of 10 mM l-phenylalanine, and water to a total volume of 3 ml. After 1 h incubation at 37°C, the reaction was stopped by the addition of 0.1 ml 1 N HCl, and the absorbance was read at 290 nm on a NANODROP 2000C spectrophotometer (Thermo Scientific, Waltham, MA). The enzyme activity was expressed in units, each representing the amount of enzyme catalyzed for the formation of 1 μM of *trans*-cinnamic acid (*ε* = 9,000 M ml^−1^) per min per milligram protein. The experiment was performed in duplicate with two flasks sampled for each repetition.

### Protein content determination.

The protein content was determined according to the Bradford method (Bradford 1976) with a standard curve prepared using bovine serum albumin. All chemicals were purchased from Sigma-Aldrich (St. Louis, MO).

### Cytotoxicity assay.

The promyelotic leukemia (HL-60) and cervical cancer (HeLa) cell lines were obtained from the Department of Medical Biotechnology of the Intercollegiate Faculty of Biotechnology, University of Gdansk, Medical University of Gdansk, Poland. The human breast adenocarcinoma (MCF-7) cell line was obtained from the Department of Microbiology, Tumor and Cell Biology, Karolinska Institute, Sweden. The HL-60 cell line was cultured in RPMI 1640 medium (Collins *et al*. 1977) supplemented with 10 % *w/v* fetal bovine serum, 2 mM glutamine, 100 units ml^−1^ penicillin, and 100 μg ml^−1^ streptomycin. The HeLa and MCF-7 cell lines were cultured in Dulbecco’s modified Eagle’s medium (Yee *et al*. 1985; Wild *et al*. 1991) supplemented with 10 % fetal bovine serum, 2 mM glutamine, 100 units ml^−1^ penicillin, and 100 μg ml^−1^ streptomycin. The cultures were maintained in a humidified atmosphere with 5 % CO_2_ at 37°C. All chemicals were purchased from Sigma-Aldrich.

The cytotoxic activity of the *A. euchroma* extracts was examined against the HL-60, HeLa, and MCF-7 cell lines utilizing the MTT (3-(4,5-dimethylthiazol-2-yl)-2,5-diphenyltetrazolium bromide) assay (Mossman 1983). This is a colorimetric assay that measures the activity of enzymes that reduce MTT, resulting in a change from yellow tetrazole to purple formazan in living cells. The investigated extracts were prepared from 28-d-old cells cultivated in MSA liquid media supplemented with 0.01, 0.1, or 1 mM PHE or without PHE (control).

The cells were seeded in 96-well microtiter plates (5 × 10^4^ cells per well) and treated for 24 h with extracts in the concentration range 0–100 μg ml^−1^. MTT (0.5 mg ml^−1^) was added and incubated for 3 h at 37°C following lysis of cells with dimethyl sulfoxide. Optical density of the formazan solution was measured at 550 nm with a plate reader (Victor, 1420 multilabel counter).

## Results and Discussion

### Effect of l-phenylalanine on cell suspension growth.

**Figure 2.**
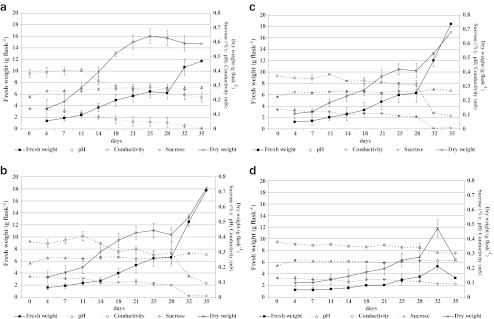
Time course of biomass increase in cell suspension culture of *A. euchroma* cultivated in MSA medium without PHE (control, *a*) or supplemented with 0.01 mM (*b*), 0.1 mM (*c*), or 1 mM (*d*) PHE. *Points* represent means ± SD, *n* = 6.

Comparison of the growth curves of *A. euchroma* cells in media containing various concentrations of PHE, or PHE-free medium as a control, is presented in Fig. 2*a*–*d*. In the control cultures and cultures supplemented with PHE, the time course of biomass accumulation was similar and steady for 4 wk from the initial subculture, with a rapid rise in biomass observed during week 5. The final increase in fresh cell biomass was measured as a ratio of final weight to initial weight. The addition of 0.01 or 0.1 mM PHE to the culture medium equally stimulated cell proliferation, resulting in a 12-fold rise in fresh cell biomass. In contrast, the cell biomass increased eightfold in control cultures and only twofold in cultures supplemented with 1 mM PHE.

Contradictory effects of PHE supplementation on cell growth have been previously reported in *in vitro* plant cultures. Masoumian *et al*. (2011) discussed the effect of PHE on biomass and secondary metabolite accumulation in different plant species. Influence of PHE on biomass depends on its concentration, applied culture medium, and plant species cultivated *in vitro*. The investigators showed that PHE supplementation (2 to 5 mg l^−1^) in *Hydrocotyle bonariensis* cultures did not significantly influence callus biomass, while in the *Artemisia absinthium* callus culture, PHE at concentration <33 mg l^−1^ showed a negative effect on cell growth.

Growth responses of *in vitro* plant cell cultures can be also monitored by determination of medium conductivity, pH, and changes in sucrose levels. In our studies, sucrose consumption of cell suspension cultures maintained in the presence of 0.01 or 0.1 mM PHE was comparable to the control cultures. The sucrose level was depleted slowly until day 28 when consumption accelerated rapidly. Sucrose levels measured on day 32 were merely 6 and 4 % of their respective baseline levels. In the untreated control cultures and cultures supplemented with 1 mM PHE, the pattern of uptake of sucrose was similar, but the sucrose content at the end of the culture period was less significantly reduced, to 16 and 70 % of the baseline values, respectively, indicative of a slower metabolic rate. The medium conductivity began to decrease from the start of the culture period, but declined faster in the untreated cultures and in the presence of 0.01 mM PHE. The pH values demonstrated a tendency to increase during the culture period in comparison to their initial levels (Fig. 2).

### Effect of l-phenylalanine on PAL activity and production of shikonin derivatives.

**Figure 3.**
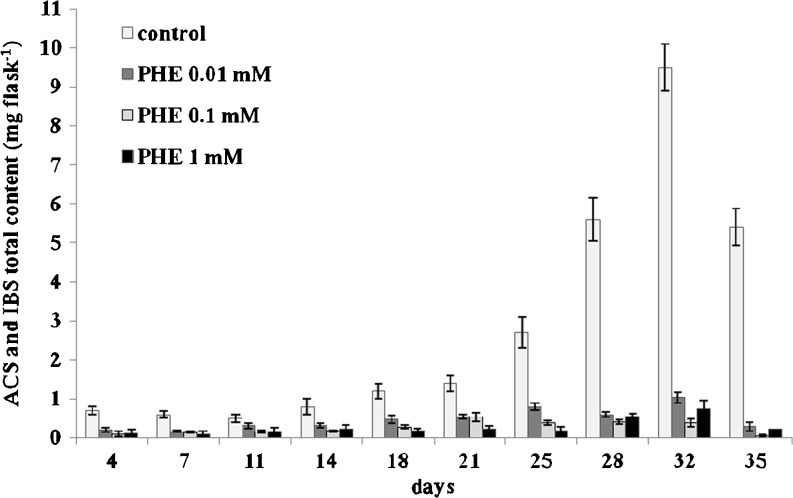
Total (intracellular + extracellular) content of ACS and IBS in suspension cultures of *A. euchroma* cultivated in MSA medium under different culture conditions. *Bars* represent means ± SD, *n* = 6.

As determined on the basis of dry mass accumulation, the highest red pigment production coincided with periods of intensive cell proliferation. The highest total (intracellular + extracellular) yield of ACS and IBS (9.5 mg per flask) was measured in the control cell suspension culture on day 32 (Fig. 3). In the presence of 0.01, 0.1, or 1 mM PHE, the maximum production of shikonin derivatives was observed on day 32 when it reached 1.0 and 0.8 mg per flask, respectively. ACS was the major component of the naphthoquinone fraction determined in cells and post-culture media. Shikonin itself was found exclusively in the post-culture media on day 32; here, cells grew in the presence of 0.01 or 0.1 mM PHE at 0.03 and 0.005 mg per flask, respectively.

The medium supplemented with PHE resulted in a decrease in ACS and IBS content compared to control cultures (Fig. 3). In comparison to control cultures, the average decrease in total ACS and IBS production was determined to be 62, 65, and 35 % in the presence of 1, 0.1, and 0.01 mM PHE, respectively. To the contrary, it has been previously reported that medium supplementation with PHE (also at doses of 1, 0.1, and 0.01 mM) had a beneficial influence on shikonin accumulation in callus cultures of *L. erythrorhizon* (Mizukami *et al*. 1977), and PHE added to the medium in *Lithospermum* cell cultures was rapidly incorporated into shikonin derivatives (Okamoto *et al*. 1995).

**Figure 4.**
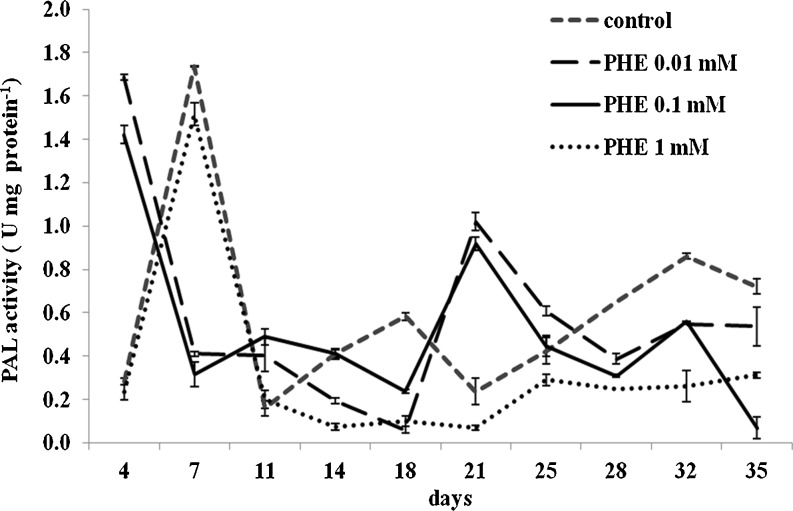
Time course of PAL activity estimated in cells cultivated under different culture conditions in MSA medium. *Bars* represent means ± SD, *n* = 4.

Under our experimental conditions, the highest PAL activity was attained in control cultures on day 7 with a second peak at day 32 (Fig. 4). Also, a transient considerable increase in PAL activity was observed as a result of treatment with 0.01 or 0.1 mM PHE on day 4 when it was 598 and 504 %, respectively, above the control values measured concurrently. Under conditions described above, the second peak in PAL activity was noted on day 21, compared to day 32 for control cultures. Only one peak of PAL activity was observed, on day 7, in 1 mM PHE-treated cultures. In *Salvia miltiorrhiza* cell cultures, PAL activity was also affected by elicitor concentration and was characterized by two peaks (Dong *et al*. 2010), which coincided with two peaks of phenolic compound accumulation. The results of these experiments are consistent with our observations (Fig. 3) where the production of shikonin derivatives in control cultures did not coincide with the first peak of PAL activity on day 7, but rather, a steady rise in accumulation was observed until 32 d which coincided with the second peak of PAL activity.

As summarized by MacDonald and D’Cunha (2007), PAL is an inducible enzyme enhanced by a wide variety of physical, chemical, and biological agents. The authors also reported that PAL kinetics is complex, controversial, highly source dependent, and not well understood overall. The first peak of PAL activity could represent a stress response of the cells when transferred to fresh medium, which is consistent with PAL activity being induced by various stress factors or elicitors (El Modafar *et al*. 2006; Sullivan 2009). Furthermore, expression of genes encoding enzymes involved in the shikonin biosynthetic pathway was stimulated in *L. erythrorhizon* cell cultures following transfer from B5 medium to the production medium M9. In this medium, the mRNA levels of PAL and other genes greatly increased within 2 h before declining rapidly and remained at low levels from 6 h to the end of the 10-d culture period (Zhang *et al*. 2010).

In earlier investigations, the temporal pattern of mRNA accumulation paralleled that of PAL enzyme activity in cell cultures of *L. erythrorhizon* (Yazaki *et al*. 1997). PAL activity was markedly induced shortly after inoculation of cells into fresh media, reaching a maximum after 24 h, then decreasing slowly to the end of the culture period. However, the rapid increase in PAL activity was closely followed by the temporary biosynthesis of benzofuran derivatives while shikonin first occurred 3 d after inoculation and continued for 2 wk. The lack of direct correlation between peaks in the production of shikonins and peaks of PAL activity under our experimental conditions may be explained by the numerous factors influencing the complex shikonin biosynthetic pathway. In *A. euchroma* cell cultures, shikonin biosynthesis was reported to be affected by medium composition, growth regulators, light regime, and pH of the medium (Zakhlenjuk and Kunakh 1998; Malik *et al*. 2011). Also, it is possible that PAL negatively inhibited *trans*-cinnamic acid production in these experiments, as has been previously reported elsewhere (MacDonald and D’Cunha 2007).

**Figure 5.**
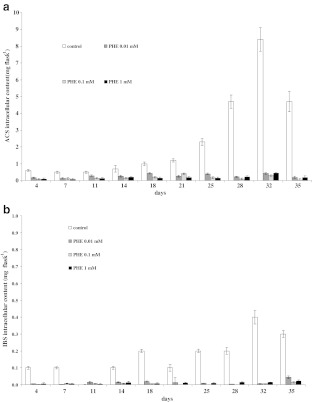
Time course of ACS (*a*) and IBS (*b*) intracellular accumulation in suspension cultures of *A. euchroma* cultivated under different culture conditions in MSA medium. *Bars* represent means ± SD, *n* = 6.

The production of intracellular ACS and IBS in the cultures supplemented with 0.01 or 0.1 mM PHE followed the same pattern until the end of the 32-d culture period when the highest yields of ACS and IBS was recorded (Fig. 5*a*, *b*). In the presence of 0.01 mM PHE, the ACS and IBS content reached 0.43 and 0.005 mg per flask, respectively. The medium supplemented with 0.1 mM PHE resulted in ACS and IBS yields of 0.3 and 0.005 mg per flask, respectively. In cells cultivated with 1 mM PHE, the highest production of ACS was 0.44 mg per flask on day 32 and that of IBS, 0.02 mg per flask on day 35 (Fig. 5*a*, *b*). However, the control cultures provided the most conducive conditions for the accumulation of shikonin derivatives, where the maximum intracellular accumulation of ACS (8.4 mg per flask) and IBS (0.4 mg per flask) was detected on day 32, being over tenfold greater than any of the supplemented cultures.

**Figure 6.**
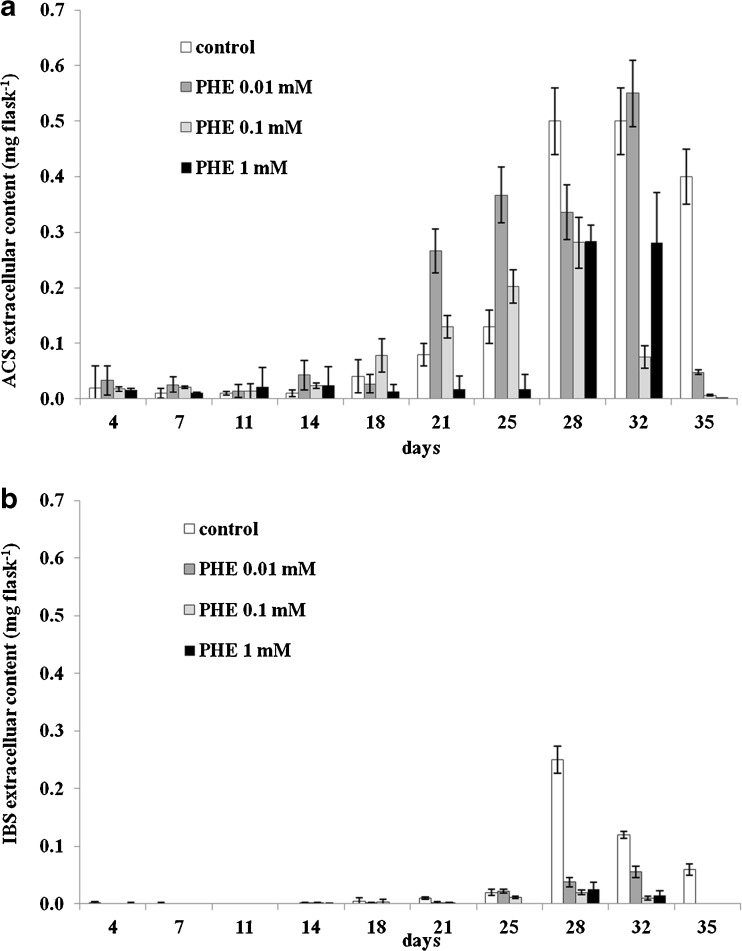
Time course of ACS (*a*) and IBS (*b*) extracellular accumulation in suspension cultures of *A. euchroma* cultivated under different culture conditions in MSA medium. *Bars* represent means ± SD, *n* = 6.

ACS and IBS were also detected in the post-culture media. While ACS was found in all the media throughout the culture period, IBS occurred during certain periods only. Similar to their intracellular levels, the highest extracellular yields of investigated compounds were found in control cultures where the maximum production attained for ACS (0.5 mg per flask) and IBS (0.25 mg per flask) occurred on days 32 and 28, respectively (Fig. 6*a*, *b*).

In the present study, the dynamics of the biomass growth and accumulation of shikonin derivatives were found to follow similar patterns, which is consistent with the results obtained by Dong *et al*. (1993) who compared these two parameters in cell suspension cultures carried out in flasks and a bioreactor.

Various strategies have been applied to enhance the production of shikonin and its derivatives in *Arnebia* sp. *in vitro* cultures. Fungal elicitors induced shikonin yields in *A. euchroma* suspension cultures, but pigment production was higher with combined fungal elicitation and *in situ* extraction (Fu and Lu 1999). Rare earth elements have been also tested for their elicitor-like effects (Ge *et al*. 2006). The cell biomass and content of shikonin derivatives were considerably enhanced in a medium supplemented with rare earth elements up to 571.1 mg l^−1^. The authors showed that the rise in cell biomass and production of shikonin-like compounds were positively correlated with the increasing PAL activity and was due to presence of rare elements.

Not only cell cultures but also hairy root cultures have been developed for shikonin production in plants of the *Arnebia* genus. *Arnebia hispidissima* was successfully transformed by *Agrobacterium rhizogenes* strain A4 (Chaudhury and Pal 2010) to produce the hairy root phenotype. Shikonin content in hairy roots growing in RC medium was estimated to be 0.85 mg g^−1^ FW at the end of the 50-d culture period.

Higher alkannin production has been achieved for an *in vitro* culture system of *A. hispidissima* compared to roots of field-grown plants (Shekhawat and Shekhawat 2011). The level of alkannin was strongly influenced by the different culture media with the highest yield of this compound recorded in cell suspension and callus cultures maintained in M9 medium.

Recently, various growth factors such as inoculum buildup, phosphate source, and *in situ* extraction methods were studied with reference to shikonin yields in cell suspension cultures of *A. euchroma* (Kumar *et al*. 2011). Direct inoculum buildup (solid to liquid), potassium phosphate sources in the medium, and *in situ* extraction with high-density paraffin significantly increased shikonin yields, up to 72 %. In our studies, PHE supplementation effectively suppressed production of shikonin derivatives, but PAL activity was not so significantly affected. PAL as a key enzyme of the phenylpropanoid pathway leads not only to shikonin biosynthesis, but also to biosynthesis of many other compounds playing essential functions in plants. It is possible that phenylalanine might be directed to the production of protectants against biotic and abiotic stress, and mechanical support (lignin) and not necessarily to the production of pigments (MacDonald and D’Cunha 2007).

### Cytotoxic activity.

The cytotoxic activity of the *A. euchroma* extracts was evaluated utilizing the MTT assay against the promyelotic leukemia (HL-60), cervical cancer (HeLa), and breast cancer (MCF-7) cell lines. The most potent cytotoxic effects were exerted by extracts prepared from the control cultures, without PHE supplementation (Table 1). These results predictably correlated with control cultures also having the highest yield of shikonin derivatives, where ACS was the major constituent. Xiong *et al*. (2009) reported that ACS, isolated from *A. euchroma* cell suspension cultures, inhibited the growth of various neoplastic cell lines in a dose-dependent manner. ACS not only was found to be most active against the Lewis lung carcinoma at a dose of 2.72 μg ml^−1^, but also inhibited the growth of MCF-7 cells at a dose of 6.82 μg ml^−1^. In this study, the highest biological activity of the total extracts prepared from control cell cultures was shown against the HL-60 cell line, with a mean inhibitory concentration (IC_50_) value of 0.8 μg ml^−1^. However, significant cell growth inhibition was also observed in cultures of MCF-7 cells (IC_50_ = 8 μg ml^−1^).

**Table 1.** The cytotoxic activity of the *A. euchroma* extracts in the concentration range of 0–100 μg ml^−1^ tested against the promyelotic leukemia HL-60, cervical cancer HeLa, and breast cancer (MCF) cell lines measured by MTT assay

l-Phenylalanine treatment	IC_50_ (μg ml^−1^)^a^		
	HL-60	HeLa	MCF-7
Control, untreated culture	0.3 ± 0.02	13 ± 0.9	8 ± 0.6
00.1 mM PHE	0.7 ± 0.03	25 ± 1.2	13 ± 0.2
0.1 mM PHE	1.4 ± 0.1	37 ± 2.2	28 ± 2.5
1 mM PHE	1.3 ± 0.1	44 ± 1.7	34 ± 3.0

^a^Means ± SD, *n* = 3

In conclusion, in comparison to control cultures, PHE failed to stimulate production of shikonin derivatives in cell suspension cultures of *A. euchroma.* The influence of PHE on biomass accumulation has been reported to depend on its concentration, applied culture medium, and plant species cultivated *in vitro.* However, the addition of PHE resulted in the modification of the red naphthoquinone excretion profile because shikonin itself was found only on day 32 in the post-culture media from cultures maintained in the presence of 0.01 or 0.1 mM PHE. It was observed that the rise in PAL activity correlated with accumulating levels of investigated naphthoquinones in control cultures. However, the highest PAL activity did not directly correlate with the highest shikonin derivative production. Under the present experimental conditions, PAL activity cannot be used as a marker of shikonin derivative production. The cytotoxic activity of the extracts obtained from the control cultures was comparable to the previously described cytotoxic activity of ACS also isolated from *A. euchroma*.
